# Impact of Oral Citicoline, Antioxidant Vitamins, and Blackcurrant Supplementation on Primary Open-Angle Glaucoma: An OCT and OCTA Study

**DOI:** 10.3390/biomedicines13061352

**Published:** 2025-05-31

**Authors:** Piera Giunta, Luca D’Andrea, Michele Rinaldi, Maria Paola Laezza, Raffaele Piscopo, Ciro Costagliola

**Affiliations:** 1 Eye Clinic, Department of Neurosciences, Reproductive and Odontostomatological Sciences, Federico II University, Via Panisini n 5, 80131 Naples, Italy; piera.giunta1@gmail.com (P.G.); michrinaldi@libero.it (M.R.); raffaele.piscopo2@unina.it (R.P.);; 2 Department of Medicine and Health Sciences “V. Tiberio”, University of Molise, 86100 Campobasso, Italy; m.laezza@studenti.unimol.it

**Keywords:** glaucoma, citicoline, blackcurrant, vitamins, OCT, OCTA

## Abstract

**Purpose:** We sought to evaluate the long-term effects of oral citicoline; vitamins A, B, C, and E; and blackcurrant therapy in patients with primary open-angle glaucoma (POAG) using optical coherence tomography (OCT), OCT angiography (OCTA), and microperimetry parameters. **Materials and Methods:** Fifteen patients with POAG (the treated group) received one soluble liquid sachet of a complementary dietary supplement containing, in a fixed combination, citicoline; vitamins A, B, C, and E; and blackcurrant (Citizin^®^, Bruschettini s.r.l., Genova, Italy) daily for 20 days a month for 1 year. Fifteen age-matched patients with POAG were given a placebo and served as a control group. The patients underwent best-corrected visual acuity (BCVA) analysis, Goldmann applanation tonometry, microperimetry examination, OCT, and OCTA at the beginning of the study and then 1, 6, and 12 months later. **Results:** A significant improvement in the overall retinal nerve fiber layer (RNFL) thickness values (compared with the control group) was recorded at the 6- (*p* < 0.009) and 12 (*p* < 0.001)-month follow-ups in the treated group. The ganglion cell complex (GCC) increased in thickness (compared with the control group) at the 12-month follow-up (*p* < 0.0001) in the treated group. The mean macular vessel density (MVD) and the mean peripapillary vessel density (PVD) in the treated group were significantly higher than those in the control group at the 12-month follow-up. Microperimetry examination, BCVA, and Goldmann applanation tonometry showed no statistically significant alterations. **Conclusions:** A fixed combination of citicoline; vitamins A, B, C, and E; and blackcurrant administered orally may have a positive impact on RNFL, GCC, MVD, and PVD in patients with POAG.

## 1. Introduction

Glaucoma comprises a heterogeneous group of optic neuropathies characterized by a progressive loss of ganglion cell complex (GCC) and typical alterations in the optic nerve head (ONH), leading to permanent visual field impairment [1]. This degeneration is primarily attributed to mechanical stress and ischemic processes induced by elevated intraocular pressure (IOP), which compromises retinal ganglion cell (RGC) viability and function.

Glaucoma, in its different subtypes (primary open-angle glaucoma (POAG); primary angle-closure glaucoma; normal-tension glaucoma; pseudoexfoliative glaucoma; etc.), represents a prominent cause of permanent vision impairment, with over 112 million individuals projected to be impacted by 2040. Age and high intraocular pressure (IOP) have been recognized as the primary risk factors. [1,2].

Although elevated IOP is considered the most crucial risk factor for POAG and remains the only modifiable factor and therapeutic target, it is essential to acknowledge that other factors come into play, influencing the course of the disease. Managing IOP alone does not completely halt the progression of glaucoma [1,2]. Nonetheless, the sole therapeutic approach presently available involves reducing IOP through hypotensive medications or surgical procedures [3].

Despite the correlation between IOP levels and GCC death, nerve damage may persist even with pressure monitoring and treatment. Glaucomatous optic neuropathy is observable even in individuals with IOP readings considered within the normal range [4]. Findings such as thinning of the neuroretinal rim, cupping, and sectoral retinal nerve fiber layer (RNFL) defects may be observed. However, research has indicated that glaucoma impacts the optic nerve and leads to degenerative alterations in the central visual pathway, lateral geniculate nucleus, and visual cortex. This degeneration cannot be solely attributed to elevated IOP, pointing to the involvement of other underlying mechanisms [5]. This suggests that there are additional factors contributing to the development and progression of glaucoma, making it a complex condition requiring comprehensive evaluation and treatment.

One such mechanism involves increased oxidative stress, as evidenced by the presence of elevated oxidative stress markers in patients with POAG and primary angle-closure glaucoma [6,7]. Additionally, studies have noted a significant correlation between oxidative DNA damage, increased IOP, and visual field defects in glaucoma patients, particularly in the human trabecular meshwork [6,7]. Further elucidating the role of oxidative stress in GCC damage and glaucoma progression is crucial for understanding the disease’s pathogenesis and developing targeted therapeutic interventions.

Given the multifactorial nature of glaucoma, researchers have explored various therapeutic agents for its management. Some of these compounds include purinergic ligands, PI3K/Akt activators, KATP channel activators, antioxidants, gases (such as nitric oxide, carbon monoxide, and hydrogen sulfide), nitric oxide synthase inhibitors, ligands for dopamine and serotonin receptors, cannabinoids, neurotrophic factors, and citicoline [8,9].

Among the various molecules studied for the treatment of glaucoma are citicoline, renowned for its neuroprotective activity, and vitamins A, B, C, and E, known for their beneficial effects on eye health and potential neuroprotective properties. Furthermore, combining blackcurrant with these molecules has been shown to have a synergistic effect in promoting an increase in retinal microcirculation, thereby improving macular perfusion and optic nerve perfusion.

Citicoline is an endogenous molecule with neuromodulatory and neuroprotective properties that plays a role in the biosynthesis of phospholipids in cell membranes and increase neurotransmitter levels in the central nervous system. Citicoline, when metabolized into cytidine and choline, plays a crucial role in the biosynthesis of cell membrane phospholipids and can increase neurotransmitter levels in the central nervous system [9]. It helps maintain essential components like sphingomyelin and cardiolipin, which might be linked to glaucoma, suggesting that citicoline could be a potential method of preventing cell death in glaucoma [10]. Studies have shown that citicoline can reduce pro-apoptotic effects and protect against synaptic loss in neural tissues [11]. Additionally, citicoline mediates neurodegenerative processes by reducing glutamate excitotoxicity [12], lessening oxidative stress [13], increasing neurotrophin levels, and improving deficits in axonal transport [14], making it particularly beneficial in protecting the vulnerable ganglion cell complex (GCC) [15].

Vitamin A plays several roles, including in immune modulation, bone remodeling, and endothelial cell maintenance, with its most critical function being visual phototransduction, the process that converts light into electrical signals [16]. A study from the NHANES involving 2912 participants found that high-dose vitamin C supplements were linked to a reduced likelihood of glaucoma, although serum vitamin C levels did not correlate with glaucoma prevalence [17]. Low levels of vitamin E in the aqueous humor have been associated with glaucoma, but studies on dietary vitamin E intake and primary open-angle glaucoma (POAG) have not shown significant associations [18]. While vitamins A, C, and E individually do not appear to protect against glaucoma, their combined use seems to lower glaucoma risk, possibly due to a synergistic antioxidant effect, as suggested by the SUN Project [16,19].

Vitamin B is essential as a REDOX cofactor and plays a crucial role in neuronal survival, particularly in axon neuroprotection. It has been recognized for its potential therapeutic effects on aging and neurodegenerative diseases [20,21,22]. Additionally, vitamin B enhances oxidative phosphorylation, buffers against metabolic stress, and increases mitochondrial size and motility, supporting its use as a neuroprotective therapy, particularly in long-term nicotinamide treatment for glaucoma patients [23].

Blackcurrants (Ribes nigrum), rich in anthocyanins (ACs), have demonstrated therapeutic potential for various conditions, including hypertension, cardiovascular diseases, neurodegenerative disorders, and ocular conditions. Oral administration of blackcurrant ACs has been shown to improve visual functions, increase blood flow in the optic nerve head (ONH), and alter plasma concentrations of endothelin-1. Additionally, blackcurrants contain compounds with antioxidant, anti-inflammatory, neuroprotective, and vasoprotective properties, making them a valuable dietary supplement [24]. To evaluate the long-term effects of these treatments in patients with POAG, we aimed to utilize optical coherence tomography (OCT) and OCT angiography (OCTA), non-invasive imaging tools that facilitate the diagnosis and monitoring of glaucoma. These techniques allow for objective measurements of the ONH and macula, along with quantitative and qualitative analysis of retinal and choriocapillaris vessel density and vascular perfusion changes [25,26].

By employing OCT, OCTA, and microperimetry parameters, we sought to comprehensively assess the potential benefits of oral citicoline; vitamins A, B, C, and E; and blackcurrant therapy in patients with POAG. Through these evaluations, we aim to shed further light on effective treatment modalities for managing glaucoma and preserving visual function in affected individuals.

## 2. Materials and Methods

### 2.1. Study Design and Intervention

This prospective study included 30 eyes of 30 patients who were followed up with a diagnosis of POAG in the Eye Clinic Federico II University between January 2022 and December 2022. For the purposes of statistical analysis, only one eye per patient (the right eye) was selected to avoid inter-eye correlation and potential bias. The sample size was calculated prior to the initiation of the study. Retinal nerve fiber layer (RNFL) thickness was the primary outcome used for this calculation. Based on data from previous studies and our own cohort, we anticipated that citicoline treatment would lead to an at least 20% improvement in RNFL thickness at the 12-month mark. To detect this change with a power of 80% and a significance level of 0.05, a minimum of 12 patients per group was required. To allow for potential dropouts, we included 15 patients per group, resulting in a total of 30 patients. The trial was a randomized, single-blind, and placebo-controlled. Patients were randomly assigned to the treatment and control groups using computer-generated random numbers to ensure unbiased allocation.

To assess the potential benefits of a specific treatment approach, the participants were divided into two groups: a treated group and a control group. The treated group received a unique combination of oral dietary supplements (Citizin^®^ sachet, Bruschettini s.r.l., Genoa, Italy), formulated in a soluble liquid sachet format for optimal absorption and convenience. This formulation included the following key components: citicoline (500 mg); vitamins A (300 μm), B group (4.415 mg), C (115 μm), and E (5 μm); and blackcurrant extract (50 mg). The total vitamin content listed includes both the purified forms added to the formulation and the naturally occurring micronutrients present in the blackcurrant extract, ensuring a consistent total dose per sachet. The participants in the treated group were instructed to take one sachet daily for 20 days each month, followed by a 10-day pause, and this therapy cycle was repeated throughout the 12-month follow-up period. The 10-day wash-out period was included each month to ensure that any residual effects of the treatment did not carry over into the next cycle. This approach was used to prevent overestimation of the treatment’s efficacy by eliminating any potential cumulative effects that could influence subsequent treatment cycles. All patients in both the treatment and placebo groups had been undergoing stable monotherapy with prostaglandin analogues (latanoprost) for at least 3 months before enrollment. No changes in hypotensive therapy were allowed during the 12-month follow-up period. This pharmacological consistency was adopted to avoid variability due to different mechanisms of action or the systemic effects of alternative hypotensive agents such as beta-blockers. The control group received an oral placebo with the same dosage as the treated group. The placebo was composed of inert ingredients, including maltodextrin, natural flavors, and colorants, designed to match the active treatment in appearance and taste without containing any active pharmacological components.

To ensure this study’s reliability and focus on a specific subset of patients, inclusion and exclusion criteria were strictly adhered to. According to the inclusion criteria, we included patients of either sex, aged between 18 and 75 years, with a confirmed diagnosis of POAG. The age range of 18 to 75 years was selected to encompass the adult population most commonly affected by POAG. This range also excludes younger patients who might present with different forms of glaucoma and older patients who may have comorbidities that could confound the study results. Patients with controlled IOP below 20 mmHg who had been undergoing the same topical hypotensive therapy for at least 3 months and had myopia corresponding to less than 5 diopters (D) were considered eligible for inclusion. Conversely, patients who had IOP levels exceeding 21 mmHg, hyper-sensitivity to citicoline, a history of optic neuritis, previously undergone glaucoma or retinal surgery, undergone prior cataract or refractive surgery, macular degeneration or other retinal disorders, or any systemic diseases that could lead to neurodegeneration (e.g., multiple sclerosis, diabetes, etc.) were excluded from the study. Moreover, patients who were on systemic medications that could significantly affect IOP values (e.g., β-blockers) were also excluded. All patients in both the treatment and placebo groups used only topical intraocular pressure-lowering medications, with no other types of eye drops being administered during the study.

### 2.2. Examinations and Outcome Measures

Clinical ophthalmologic examinations were meticulously performed at the onset of the study and continued later at 1, 6 and 12 months. These examinations included a best corrected visual acuity (BCVA) exam, IOP measurements using Goldmann applanation tonometry, and slit-lamp dilated fundus examination.

Measurements of retinal ganglion cell thickness, RNFL, macular vessel density (MVD), and peripapillary vessel density (PVD) were performed using OCT and OCTA (Angiovue, Optovue, Inc., Fermont, CA, USA). OCTA is based on a split-spectrum amplitude-decorrelation angiography algorithm for which blood flow is used as the intrinsic contrast [27]. Specifically, a 6.0 × 6.0 mm^2^ area centered on the foveal region and a 4.5 × 4.5 mm^2^ area on the optic nerve were examined to measure the MVD and the PVD, respectively. The OCTA device was employed along with the 3-dimensional (3D) projection artifact removal (PAR) algorithm to remove projection artifacts for improving depth resolution in an OCTA signal and then distinguishing vascular plexus-specific features [25,28]. Scans were discarded if they were of poor quality, had a signal strength index of less than 80, or had incorrect segmentation, motion artifacts, or low centration. Retinal ganglion cell thickness and microvascular parameters were measured using standardized protocols, and interobserver reliability was assessed to ensure the accuracy and reproducibility of measurements. Interobserver reliability for OCT and OCTA measurements was assessed using the intraclass correlation coefficient (ICC), which demonstrated a high level of agreement between observers (ICC = 0.95).

Retinal sensitivity was assessed via microperimetry examination (MP-1, Nidek Technologies s.r.l., Vigonza, Italy, NAVIS software version), to which patients in both groups were subjected after being administered tropicamide (1%). The 19°, 10 dB glaucoma test was used, with a threshold strategy of 4-2, Goldmann III stimulus, and single cross 3°, recording retinal sensitivity at 24 points in the central 10° around fixation. During the examination, patients were instructed to focus their gaze on a central cross used as the fixation target. To ensure the reliability of the test results, false-positive responses were monitored using a stimulus projected onto the optic disc. The integrated software generated a color-coded map, which was utilized for analysis and description. In this map, an absolute defect, indicated by the color red, meant that patients showed no response even at an attenuation level of 0 dB. A moderate defect, indicated by the color orange, represented a response of between 2 and 6 dB. Lastly, a mild defect, shown as yellow, represented a response of 8–10 dB.

The initial clinical and paraclinical evaluations (T0) were carried out before the treatment regimen was initiated. Subsequent evaluations were scheduled to be performed at 1 month (T1), 6 months (T2), and 12 months (T3) after the commencement of the study. This systematic approach allowed for the close monitoring of the patients’ progress and provided valuable data on the long-term effects of the treatment administered.

### 2.3. Statistical Analysis

Data analysis was performed using SPSS v29 software (SPSS, Inc., Chicago, IL, USA). Comparison between the study groups was performed using a one-way analysis of variance. Changes in IOP, MVD, and PVD, as well as mean defect and mean sensitivity values, were analyzed. Paired data t-tests were used to compare values between the two groups at baseline and subsequent follow-ups (1, 6, and 12 months). A *p*-value < 0.05 was considered statistically significant.

## 3. Results

We analyzed a group of 30 patients (18 males and 12 females) with an age range of between 33 and 68 years, with a mean of 53.00 ± 7.68 years. These patients were separated into two groups, and 15 patients were randomly placed in the treated group, whose members were treated with oral dietary supplementation, while the remaining 15 patients served as the control group. Throughout the follow-up period, none of the patients in either group had an IOP exceeding 20 mmHg. The patients who received oral nutritional supplements had greater total RNFL thickness measurements than those who did not receive citicoline treatment. Surprisingly, the differences were significant during the 6-month evaluation (*p* = 0.011) and even more pronounced in the 12-month follow-up (*p* = 0.0005). Conversely, the control group registered a statistically notable decrease in RNFL thickness, in comparison to the baseline measurements, at the 12-month follow-up (*p* = 0.017).

Regarding GCC thickness, there was a notable increase in the treated group throughout the 12-month follow-up (*p* = 0.016), indicating the positive effect of citicoline supplementation. In contrast, this shift failed to reach statistical significance in the control group.

In addition, the mean microvascular density (MVD) and peripheral vascular density (PVD) in the intervention group were significantly higher at the 6-month (*p* = 0.001 and *p* = 0.0001, respectively) and 12-month (*p* = 0.002 and *p* = 0.00001, respectively) follow-ups compared to the baseline measurements. Conversely, these changes did not reach statistical significance in the control group.

**Figure 1:**
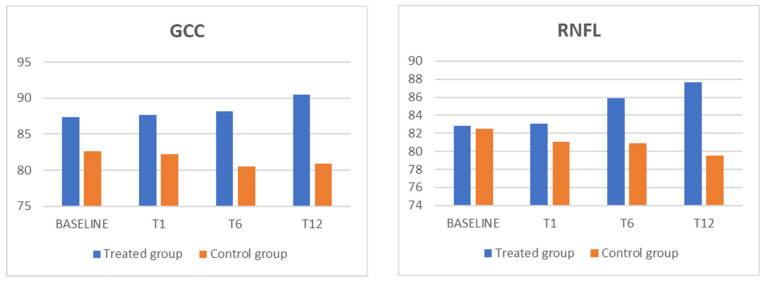
The GCC and RNFL parameters for showed a statistically significant increase, in comparison with the control group, in the treated group. GCC: ganglion cell complex; RNFL: retinal nerve fiber layer.

**Figure 2:**
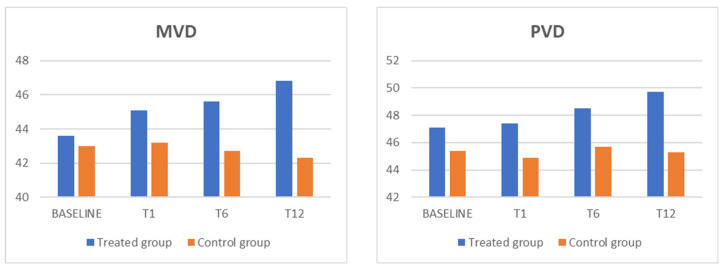
The MVD and PVD parameters showed a statistically significant increase, in comparison with the control group, in the treated group. MVD: macular vessel density; PVD: peripapillary vessel density.

When comparing the mean defect and mean sensitivity values obtained via microperimetry examination, no statistical change was observed during the follow-up period in either group. In addition, BCVA and IOP measurements remained stable and showed no significant changes during the follow-up period. Table 1 and Table 2 and Figure 1 and Figure 2 detail the actual data and results pertaining to these measurements and comparisons.

Throughout the duration of the study, the patients were monitored for any potential side effects related to the therapy. No significant side effects were reported by the patients in either the treatment or placebo groups. The therapy was generally well tolerated, with all the patients completing the full course without reporting any adverse events requiring discontinuation or additional medical intervention.

## 4. Discussion

Glaucoma is a leading cause of irreversible blindness worldwide, characterized by the progressive degeneration of the ganglion cell complex (GCC) and the optic nerve. This multifactorial disease is most commonly associated with elevated intraocular pressure (IOP), which is the primary modifiable risk factor. However, many patients continue to experience disease progression despite well-controlled IOP, highlighting the involvement of additional pathological mechanisms in glaucomatous optic neuropathy [14,29,30,31].

Beyond IOP, factors such as oxidative stress, impaired microcirculation, and neurotrophic factor deprivation have been implicated in the ongoing degeneration of retinal ganglion cells [32]. These insights suggest that comprehensive treatment strategies targeting these additional pathways may be necessary to prevent further vision loss [33].

Recent research has focused on the potential benefits of combining neuroprotective agents and antioxidants with traditional IOP-lowering therapies. Among the most promising compounds are citicoline, which supports neuronal membrane integrity and neurotransmitter function, and a variety of vitamins and natural extracts, such as vitamins A, B, C, and E and blackcurrant extract, which have demonstrated synergistic antioxidant and neuroprotective effects [10,11,12,13,14,17,18,19,23].

To assess the effectiveness of additional treatments, it is essential to conduct an evaluation based on clinical evidence, which should rely on standard objective parameters and be validated using well-established measurement tools. In the context of glaucoma, there are several essential gold standards for assessing neuroprotection and visual function, including perimetry (visual field testing), OCT, and OCTA.

In this study, we combined all these molecules with regular ocular hypotensive medication in the context of glaucoma patients. The treatment regimen involved administering this dietary supplement for 20 days, followed by a 10-day interruption every month, over a duration of 12 months. Throughout the study, we utilized perimetry, OCT, and OCTA as measurement tools to evaluate the potential benefits of citicoline; vitamins A, B, C, and E; and blackcurrant extract as a treatment modality for glaucoma.

A study conducted by Ohguro et al. [34]. reported that administering blackcurrant (50 mg/day) systemically to patients with primary open-angle glaucoma (POAG) (*n* = 30) for 6 months resulted in a significant increase in blood flow at the optic nerve head (ONH) (*p* < 0.05). Furthermore, in a 24-month double-masked, placebo-controlled trial, the authors demonstrated that patients with POAG who were administered blackcurrant extract showed less worsening of mean deviation and increased ocular blood flow compared to the placebo-treated patients during the 24-month trial period [35].

According to Ohguro et al. [34], our results reveal a significant improvement in MVD and PVD among the treated patients (*p* < 0.01) compared to the control group after 12 months of treatment. This enhancement in perfusion is primarily attributed to the vaso-protective activity of blackcurrant ACs, which have been shown to increase blood flow.

Furthermore, the results show a significant improvement in RNFL and GCC thickness among the treated patients (*p* < 0.01) compared to the control group after 12 months of treatment. This notable enhancement can be attributed to several factors. Firstly, the improvement in blood flow observed in the treated patients enhanced perfusion at the ONH, thus contributing to the improvement in RNFL thickness and GCC. The neuroprotective and neuroregenerative effects of citicoline, combined with the synergistic antioxidant effect of vitamins A, C, and E, also contributed positively to this improvement. Additionally, the reduction in oxidative stress mediated by vitamin B had a beneficial impact. Finally, the blackcurrant’s ability to neutralize free radicals and oxidizing molecules produced by cellular metabolism played a significant role. However, it is important to recognize that while RNFL thickness is a valuable marker for detecting early and moderate stages of glaucoma, its effectiveness may diminish in regard to advanced-stage glaucoma. As noted by Geng W et al. [36], alternative markers or imaging modalities may be more appropriate for monitoring disease progression in such advanced cases. This limitation should be considered when interpreting the results of this study and in the design of future research.

Interestingly, when examining the mean defect and mean sensitivity data obtained via microperimetry examination, no statistically significant changes were observed during the follow-up period in either group. This indicates that the improvements in RNFL thickness and microvascular parameters in the treated group did not directly translate into significant functional changes as measured via microperimetry. In fact, microperimetry is considered a valid complementary test, but the visual field analyzed is limited, representing, in turn, a limitation in the analysis of central damage caused by glaucoma.

We believe that the protective effect at the vascular level can be attributed mainly to the presence of blackcurrant extract in the formulation. Blackcurrant is renowned for being an excellent source of polyphenols, particularly ACs, as well as micronutrients and dietary fiber. The beneficial effects of these compounds are attributed to several underlying mechanisms, including the upregulation of endothelial nitric oxide synthase, decreased carbohydrate-digestive enzyme activity, reduced oxidative stress, and inhibition of inflammatory gene expression [37,38].

In terms of visual function, berries have demonstrated positive effects such as im-proving dark adaptation and temporary refractive alteration in healthy human volunteers using video display terminals. Moreover, they have been found to aid in reducing the progression of glaucoma neuropathy [39]. Although the specific mechanisms behind these beneficial ocular effects remain unknown, it is believed that upon oral administration, the intact forms of blackcurrants are absorbed and transported beyond both the blood–aqueous barrier and the blood–retina barrier into various ocular tissues, including the choroid, retina, and ciliary body, thereby exerting beneficial biological effects [40].

Based on these data, OCT and OCTA appear to be valid non-invasive tools for detecting the neuroprotective effect of oral citicoline treatment. The findings of this study agree with those reported in other published studies [19,23,34,35], adding an analysis of microvascular changes in the macular and peripapillary retinal area using OCTA.

One of the limitations of this study is the heterogeneity of the glaucoma stages in the enrolled patients, as this study was not limited to middle-stage glaucoma. However, this characteristic provides a more realistic representation of the patient population typically encountered in glaucoma units. We suppose that early glaucoma treatment may have better and longer-lasting effects than the treatment of more severe glaucoma, even though, in practice, most glaucoma patients have already suffered severe GCC damage at the time of diagnosis. In this regard, pilot studies aimed at the early detection of glaucoma through advanced imaging and quality-of-life assessment might be helpful. Another limitation of this study is the lack of a dietary intake assessment, as diet could influence systemic vitamin levels. However, the randomized, placebo-controlled design helped mitigate this potential bias across treatment groups. Future studies can incorporate dietary records or serum vitamin assays to further address this variable. To minimize potential sources of bias, blinding procedures were implemented during data collection and analysis, and standardized measurement protocols were followed. Future studies should explore dose–response relationships and treatment duration effects, conduct subgroup analyses based on disease severity, and investigate the long-term safety and efficacy of the combined treatment approach. Additionally, further research elucidating the neuroprotective and vascular effects of citicoline, vitamins, and blackcurrant can provide valuable insights into the pathogenesis of glaucoma and guide the development of targeted therapeutic strategies. Moreover, considering the potential synergistic effects of the combined treatment, it would be beneficial to explore various combinations of these compounds and their dosages to optimize their therapeutic efficacy.

In conclusion, although larger studies are necessary to confirm and further explore the potential benefits of the combined treatment, the findings of this study highlight the potential of combining oral citicoline; vitamins A, B, C, and E; and blackcurrant extract as a supplementary therapeutic approach for glaucoma. This treatment modality has shown promising results in improving RNFL and GCC thickness and vascular perfusion, contributing to the preservation of visual function in patients with POAG. Further research and clinical trials would help solidify these findings and establish this combined therapy as a valuable addition to glaucoma management strategies.

## Figures and Tables

**Table 1:** Analysis of the parameters for the treated group over the follow-up periods.

	RNFL µm (Average)	GCC µm (Average)	MVD	PVD
Baseline	82.8 ± 15.9	87.4 ± 18.6	43.6 ± 6.0	47.1 ± 7.9
T1	83.1 ± 16.0	87.7 ± 18.5	45.1 ± 5.6	47.4 ± 8.2
T6	85.9 ± 16.6	88.2 ± 18	45.6 ± 5.7	48.5 ± 8.4
T12	87.7 ± 17.1	90.5 ± 18.5	46.8 ± 6.0	49.7 ± 8.5
*p*-value T0–T1	0.127	0.525	0.009	0.085
*p*-value T0–T2	0.011	0.489	0.001	0.0001
*p*-value T0–T3	0.0005	0.016	0.002	0.00001

Statistical analysis of the differences between evaluated parameters from baseline to 12-month follow-up. Data are given as means ± standard deviations according to the normality of distribution. GCC: ganglion cell complex; RNFL: retinal nerve fiber layer; MVD: macular vessel density; PVD: peripapillary vessel density; T1: 1-month citicoline treatment; T6: 6-month citicoline treatment; T12: 12-month citicoline treatment.

**Table 2:** Analysis of the parameters for the control group over the follow-up periods.

	RNFL µm (Average)	GCC µm (Average)	MVD	PVD
Baseline	82.5 ± 14.9	82.6 ± 14.5	43.0 ± 5.8	45.4 ± 6.2
T1	81.1 ± 13.4	82.2 ± 14.5	43.2 ± 5.3	44.9 ± 6.1
T6	80.9 ± 13.1	80.5 ± 14.6	42.7 ± 5.2	45.7 ± 7.9
T12	79.5 ± 13.4	80.9 ± 13.9	42.3 ± 5.2	45.3 ± 7.9
*p*-value T0–T1	0.1	0.1	0.8	0.02
*p*-value T0–T2	0.22	0.02	0.79	0.88
*p*-value T0–T3	0.017	0.24	0.48	0.94

Statistical analysis of the differences between evaluated parameters from baseline to 12-month follow-up. Data are given as means ± standard deviations according to the normality of distribution. GCC: ganglion cell complex; RNFL: retinal nerve fiber layer; MVD: macular vessel density; PVD: peripapillary vessel density; T1: 1-month placebo; T6: 6-month placebo; T12: 12-month placebo.

## Data Availability

Data are available on request.
